# Regulation of boar sperm functionality by the nitric oxide synthase/nitric oxide system

**DOI:** 10.1007/s10815-019-01526-6

**Published:** 2019-07-19

**Authors:** Florentin-Daniel Staicu, Rebeca Lopez-Úbeda, Jon Romero-Aguirregomezcorta, Juan Carlos Martínez-Soto, Carmen Matás Parra

**Affiliations:** 1 Department of Physiology, Veterinary Faculty, University of Murcia, International Excellence Campus for Higher Education and Research (Campus Mare Nostrum), Murcia, Spain; 2 Institute for Biomedical Research of Murcia (IMIB), Murcia, Spain; 3 Department of Cell Biology and Histology, Faculty of Medicine, University of Murcia, International Excellence Campus for Higher Education and Research (Campus Mare Nostrum), Murcia, Spain; 4 Department of Physiology, Faculty of Medicine and Nursing, University of the Basque Country (UPV/EHU), Bizkaia, Spain; 5 IVI-RMA Global, Murcia, Spain

**Keywords:** Nitric oxide, Nitric oxide synthase, Spermatozoa, Capacitation, In vitro fertilization

## Abstract

**Purpose:**

Nitric oxide (NO) is a free radical synthesized mainly by nitric oxide synthases (NOSs). NO regulates many aspects in sperm physiology in different species. However, in vitro studies investigating NOS distribution, and how NO influences sperm capacitation and fertilization (IVF) in porcine, have been lacking. Therefore, our study aimed to clarify these aspects.

**Methods:**

Two main experiments were conducted: (i) boar spermatozoa were capacitated in the presence/absence of S-nitrosoglutathione (GSNO), a NO donor, and two NOS inhibitors, N^G^-nitro-L-arginine methyl ester hydrochloride (L-NAME) and aminoguanidine hemisulfate salt (AG), and (ii) IVF was performed in the presence or not of these supplements, but neither the oocytes nor the sperm were previously incubated in the supplemented media.

**Results:**

Our results suggest that NOS distribution could be connected to pathways which lead to capacitation. Treatments showed significant differences after 30 min of incubation, compared to time zero in almost all motility parameters (*P* < 0.05). When NOSs were inhibited, three protein kinase A (PKA) substrates (~ 75, ~ 55, and ~50 kDa) showed lower phosphorylation levels between treatments (*P* < 0.05). No differences were observed in total tyrosine phosphorylation levels evaluated by Western blotting nor in situ. The percentage of acrosome-reacted sperm and phosphatidylserine translocation was significantly lower with L-NAME. Both inhibitors reduced sperm intracellular calcium concentration and IVF parameters, but L-NAME impaired sperm ability to penetrate denuded oocytes.

**Conclusions:**

These findings point out to the importance of both sperm and cumulus-oocyte-derived NO in the IVF outcome in porcine.

**Electronic supplementary material:**

The online version of this article (10.1007/s10815-019-01526-6) contains supplementary material, which is available to authorized users.

## Introduction

Several reactive oxygen species (ROS), including hydrogen peroxide, superoxide anion, and NO, have been shown to be involved in processes important for sperm physiology. Under normal, tightly regulated physiologic conditions, these ROS are essential for the sperm to acquire the fertilizing ability [1]. At physiologic levels, NO has been demonstrated to modulate sperm capacitation and acrosome reaction, and sperm motility, and it may also have an anti-apoptotic effect (reviewed by [2]). Besides, the importance of NO in oocyte maturation and subsequent fertilization has also been revealed [3].

It is known that sperm can produce NO, but the evidence that the endogenous synthesis is sufficient to be physiologically significant is equivocal [4]. Various cell types in the mammalian female reproductive tract generate substantial levels of NO, which in turn determine the S-nitrosylation of sperm proteins. Thus, in vivo is more likely to occur as a response to the NO generated by the female tract cells, rather than by autocrine effects of sperm-generated NO. Furthermore, it has been demonstrated that activity of NOSs, the enzymes responsible for NO synthesis, can be modulated by sexual hormones [5]; therefore, the NO levels will vary during the estrus cycle [6] which in turn could regulate the fertilization process.

Sengoku et al. [7] showed that low concentrations of NO may have a physiologic role in fertilization by enhancing the capacitation and binding to the zona pellucida (ZP), but not by inducing the acrosome reaction or facilitating oocyte penetration. On the other hand, Herrero et al. [8] showed that the incubation of spermatozoa with NOS inhibitors reduced the IVF outcome in mouse. These authors observed that NOS inhibition during sperm capacitation impaired the spontaneous acrosome reaction, as well as the IVF. However, studies on the production of certain substances during the interaction of gametes that affect IVF performance have been scarce. In this sense, it has been described that both spermatozoa and cumulus cells produce NO and this molecule takes part in the fertilization process [3, 9]. Nevertheless, despite all the studies carried out to determine the role of NO on sperm function, we should improve our understanding of how this gas modulates it by performing tests that bring us closer to the physiological conditions during fertilization. In relation to the studies using human spermatozoa and their interaction with the female gamete, it has only been possible to analyze hemizone binding assays [7, 10], logically for ethical reasons. On the other hand, IVF assays performed in mouse were done with epididymal spermatozoa which cannot be considered physiologically mature. Therefore, these studies, despite the important information they provide, cannot be considered conclusive.

It appears that while NO synthesis in sperm is required for IVF, the free radicals generated in the medium, including NO, could be in excess and be harmful, as seen in certain infertility cases [11]. In porcine, they could affect the functionality of both spermatozoa and oocytes and, somehow, contribute to the problem of polyspermy (i.e., fertilization of an ovum by more than one spermatozoon) in this species. However, polyspermy could be used as a tool to evaluate sperm functionality since a higher percentage of penetrated oocytes and sperm number per penetrated oocyte correlate with sperm quality [12].

For all the reasons above, this paper aims to determine the role of the NOS/NO system in the fertilizing capacity of boar spermatozoa. Besides, since the NO function during the fertilization process in porcine has not yet been determined, we hypothesized that by regulating the NOS/NO system, the IVF efficiency could be improved. To develop this hypothesis, we determined, at first, the NO effects on the spermatozoon, followed by its impact on the IVF.

## Materials and methods

### Ethics

The study was carried out following the Spanish Policy for Animal Protection RD 53/2013, which meets European Union Directive 2010/63/UE on animal protection. The Ethics Committee of Animal Experimentation of the University of Murcia and the Animal Production Service of the Agriculture Department of the Region of Murcia (Spain) (ref. no. A13160609) approved the procedures performed in this work.

### Materials

Unless otherwise stated, chemicals and reagents were purchased from Sigma-Aldrich Química S.A. (Madrid, Spain). Equine chorionic gonadotropin (eCG; Foligon) was supplied by Intervet International B.V. (Boxmeer, Holland), human chorionic gonadotropin (hCG; Veterin Corion) by Divasa-Farmavic (Barcelona, Spain), and Percoll by GE Healthcare (Uppsala, Sweden). The prolonged anti-fade mounting medium (SlowFade Antifade Kit) was obtained from Invitrogen (Paisley, UK). N^G^-nitro-L-arginine methyl ester (L-NAME; 483125) was purchased from Calbiochem (distributed by Merck Chemicals, Beeston, Nottingham, UK).

### Culture media

In vitro maturation (IVM) of pig oocytes was carried out using the NCSU-37 medium [13] supplemented with 0.57 mM cysteine, 1 mM dibutyryl-cAMP, 5 mg/mL insulin, 50 μM β-mercaptoethanol, 10 IU/mL eCG, 10 IU/mL hCG, and 10% *v/v* porcine follicular fluid.

Sperm capacitation and IVF were performed using Tyrode’s albumin lactate pyruvate (TALP) medium [14], consisting of 114.06 mM NaCl, 3.2 mM KCl, 8 mM Ca lactate·5H_2_O, 0.5 mM MgCl_2_·6H_2_O, 0.35 mM NaH_2_PO_4_, 25.07 mM NaHCO_3_, 10 mM Na lactate, 1.1 mM Na pyruvate, 5 mM glucose, 2 mM caffeine, 3 mg/mL bovine serum albumin (BSA, A-9647), 1 mg/mL polyvinyl alcohol (PVA), and 0.17 mM kanamycin sulfate.

### Sperm collection

Sperm samples were collected from boars with proven fertility by the gloved hand method. Standard laboratory techniques were applied to evaluate sperm concentration, motility, acrosome integrity, and normal morphology.

### Immunocytochemistry: NOS detection and Tyr-P by IIF

To determine NOS localization, a method adapted from Meiser and Schulz [15] was used. Briefly, ejaculated boar sperm were washed with Dulbecco’s phosphate-buffered saline without calcium chloride and magnesium chloride (DPBS) and spread on glass slides coated with poly L-lysine. Spermatozoa were air-dried and fixed for 20 min in ice-cold 3% *v/v* paraformaldehyde in DPBS containing 120 mM sucrose. They were gently rinsed with DPBS, incubated for 10 min in ice-cold 100% *v/v* methanol, and triply washed with DPBS. Specimens were treated with blocking I solution (10% *w/v* BSA, 1% *v/v* Triton X-100, dissolved in distilled water, 1 h, 20 °C). Next, sperm were incubated with blocking II solution (2% *w/v* BSA, 1% *v/v* Triton X-100, dissolved in distilled water, 1 h, 37 °C), which included the primary anti-NOS antibodies (all three produced in mouse, 1:1000): anti-nNOS (N2280, monoclonal, clone NOS-B1, obtained with a recombinant nNOS fragment [amino acids 1–181] from rat brain), anti-eNOS (N9532, monoclonal, clone NOS-E1, obtained with a synthetic peptide corresponding to bovine eNOS [amino acids 1185–1205 with an N-terminally added lysine] conjugated to keyhole limpet hemocyanin [KLH]), or anti-iNOS (N9657, monoclonal, clone NOS-IN, obtained with a synthetic peptide corresponding to iNOS from mouse macrophage [amino acids 1126–1144] conjugated to KLH). These anti-NOS antibodies were chosen since their reactivity with porcine sperm extracts was previously shown by Aquila et al. [16]. Then, the specimens were triply washed with blocking II and probed overnight (4 °C) with a FITC-labeled secondary antibody (goat anti-mouse, 1:1000, diluted in blocking II). For controls, specimens were processed in the absence of primary and/or secondary antibody.

Tyrosine phosphorylation (Tyr-P) location was studied as previously described [17], using an anti-phosphotyrosine antibody (4G10, Millipore, CA, USA, 1:300 in 1% *w/v* BSA). The secondary antibody was a fluorescein-conjugated goat anti-mouse (Bio-Rad Laboratories, Madrid, Spain, 1:400 in 1% *w/v* BSA).

All images were taken at ×1000 (for NOS distribution) and ×400 (for Tyr-P location) magnifications, using the AxioVision Imaging System (Rel. 4.8) with an AxioCam HRc camera (Carl Zeiss, Göttingen, Germany) attached to a Leica DMR fluorescence microscope (Leica Microsystems, Wetzlar, Germany) equipped with a fluorescent optical blue filter (BP 480/40; emission BP 527/30).

### Spermatozoa motion assay

To evaluate sperm motility, computer-assisted sperm analysis (CASA) was performed (ISAS® system, PROiSER R+D S.L., Valencia, Spain), and the following parameters were studied: total motility (%), progressive motility (%), curvilinear velocity (VCL, μm/s), straight-line velocity (VSL, μm/s), average path velocity (VAP, μm/s), linearity of the curvilinear trajectory (LIN, ratio of VSL/VCL, %), straightness (STR, ratio of VSL/VAP, %), amplitude of lateral head displacement (ALH, μm), wobble of the curvilinear trajectory (WOB, ratio of VAP/VCL, %), and beat cross-frequency (BCF, Hz). For this purpose, a 4-μL drop of the sample was placed on a warmed (38.5 °C) Spermtrack ST20 chamber (PROISER R+D S.L) and analyzed using a phase-contrast microscope (×200 magnification; Leica DMR, Wetzlar, Germany). The setting parameters were 60 frames at 30 frames/s, of which spermatozoa had to be present in at least 15 to be counted. Spermatozoa with a VCL less than 10 μm/s were considered immotile. A minimum of five fields per sample were evaluated, counting a minimum of 200 spermatozoa per field.

### Western blotting: PKAs-P and Tyr-P

Sperm protein extracts were isolated from 1 × 10^6^ spermatozoa/sample and immunoblotted as described by Navarrete et al. [18] with the following antibodies: anti-phospho-PKA substrates (9624, Cell Signaling Technology, Beverly, USA, 1:2000), anti-phosphotyrosine (4G10, Millipore, CA, USA, 1:10000), and anti-β-tubulin (T0198, Sigma-Aldrich®, Madrid, Spain, 1:5000). The Pierce® ECL 2 Western Blotting Substrate (80196, Lumigen Inc., Southfield, MI, USA) coupled with a chemiluminescence system (Amersham Imager 600, GE Healthcare Life Sciences, Buckinghamshire, UK) were used to visualize the blots. The relative amount of signal in each membrane was quantified using the ImageQuant TL v8.1 software (GE Healthcare).

### Acrosome reaction assay

Boar spermatozoa were capacitated for 1 h and subsequently exposed for 30 min to 3 ng/mL progesterone under different experimental conditions, after which the percentage of acrosome-reacted sperm was evaluated by staining with FITC-conjugated peanut agglutinin from *Arachis hypogaea* (PNA-FITC L7381, Sigma-Aldrich®, Madrid, Spain), as previously described [19]. Samples were analyzed under an epifluorescence microscope at ×400 magnification.

### Detection of membrane PS translocation

Translocation of phosphatidylserine (PS) residues to the outer leaflet of the plasma membrane was detected with an Annexin V-Cy3™ Apoptosis Detection Kit (Sigma, Madrid, Spain). For this assay, 1 μL Annexin V with 5 μL 6-carboxyfluorescein diacetate (6-CFDA) in 450 μL of binding buffer (commercial kit) was mixed with 50 μL of each sperm sample. After 10 min of incubation in the dark, at room temperature, samples were fixed with 10 μL formaldehyde (10% *v/v* in DPBS). Each sample was placed on a slide and examined at ×400 magnification by epifluorescence microscopy. Viable sperm (6-CFDA+) were visualized in green with a standard fluorescein filter and Annexin+ sperm (labeling PS exposure, Annexin V-Cy3.18+) in red (N2.1 filter; excitation BP 515–560 nm) [20].

### Determination of [Ca^2+^]_i_

Intracellular calcium concentration ([Ca^2+^]_i_) was measured according to a method reported previously [21, 22]. Specifically, spermatozoa were incubated with 2.5 μM Fura-2/AM in a buffer medium consisting of 2.7 mM KCl, 1.5 mM KH_2_PO_4_, 8.1 mM Na_2_HPO_4_, 137 mM NaCl, 5.55 mM glucose, and 1 mM pyruvate for 45 min at 37 °C. The extracellular unloaded Fura-2 was removed by centrifugation (700×*g*, 5 min). Washed sperm were resuspended in the same buffer to a concentration of 3 × 10^8^ cells/mL and incubated at 37 °C for 15 min in the dark. Then, spermatozoa were centrifuged (700×*g*, 5 min) and resuspended in TALP medium. As a negative control, spermatozoa were also resuspended in DPBS. Fluorescence was monitored using the Jasco FP-6300 spectrofluorometer (Jasco, Madrid, Spain) for a further 30 min. Excitation wavelengths alternated between 340 and 380 nm with emission held at 510 nm. At the end of the experiments, sperm were lysed with 0.5% *v/v* Triton X-100, and then Ca^2+^ was depleted by addition of 25 mM EGTA. [Ca^2+^]_i_ was calculated as previously described [23]. For the statistical analysis, the Ca^2+^ concentration (nM/L) was recorded from 0 to 1800 s at 30-s intervals for every experimental group and replicate. Finally, the mean value during the incubation period was calculated.

### Oocyte collection and IVM

Ovaries from Landrace by Large White gilts were collected at a local slaughterhouse (El Pozo Alimentación S.A., Alhama de Murcia, Murcia, Spain) and transported within 30 min after slaughter to the laboratory in saline solution containing 100 μg/mL kanamycin sulfate at 38.5 °C. Before collecting the cumulus-oocyte complexes (COCs), ovaries were washed once in 0.04% *w/v* cetrimide solution and twice in saline. COCs from antral follicles (3–6 mm diameter) were washed twice with DPBS supplemented with 1 mg/mL PVA and 0.005 mg/mL red phenol, and twice more in maturation medium previously equilibrated for a minimum of 3 h at 38.5 °C under 5% CO_2_ in air. Groups of 50 COCs with complete and dense cumuli oophori were cultured in 500 μL maturation medium for 22 h at 38.5 °C under 5% CO_2_ in air. Following this incubation, COCs were washed twice in fresh maturation medium without dibutyryl cAMP, eCG, and hCG and cultured for an additional period of 20–22 h.

### IVF and zygote staining

Following the 44 h culture in maturation medium, COCs were stripped or not (see “Experimental design”) of cumulus cells by pipetting and then washed three times with TALP medium. The IVF medium was previously equilibrated at 38.5 °C under 5% CO_2_ in a four-well dish (250 μL/well), and groups of 50 oocytes were transferred into each well. Semen aliquots (0.5 mL) from different boars were mixed and subjected to a discontinuous Percoll gradient (45 and 90% *v/v*, 740×*g*, 30 min). The resultant sperm pellets were diluted in TALP medium and centrifuged again (10 min at 740×*g*). After diluting the pellet again in TALP, an aliquot of this suspension was used for IVF, giving a final concentration of 2.5 × 10^5^ spermatozoa/mL or 2.5 × 10^4^ spermatozoa/mL, depending on the experiment. The IVF medium was supplemented with NOS inhibitors or NO donor or not supplemented, as described in the experimental design. At 18–20 h post-insemination, putative zygotes were fixed and stained for evaluation as previously described [3].

### Statistical analysis

The data are presented as the mean ± standard error of the mean (SEM) and were tested for normality using the Kolmogorov-Smirnov test, and the homogeneity of variance was determined using the Levene test. ANOVA was used for the statistical analysis, and the means were separated using the Tukey test at *P* < 0.05. Since the data regarding the acrosome reaction experiment did not satisfy the Kolmogorov-Smirnov and Levene tests, the Kruskal-Wallis test was applied, and treatment average ranks were separated using the stepwise step-down multiple comparisons method [24] at *P* < 0.05. The true means of the data, rather than ranked means, are presented. All statistical analyses were conducted using IBM SPSS Statistics for Windows, Version 20.0 (IBM, Armonk, NY, USA).

### Experimental design

#### Experiment 1: effects of NO on sperm capacitation

**Fig. 1:**
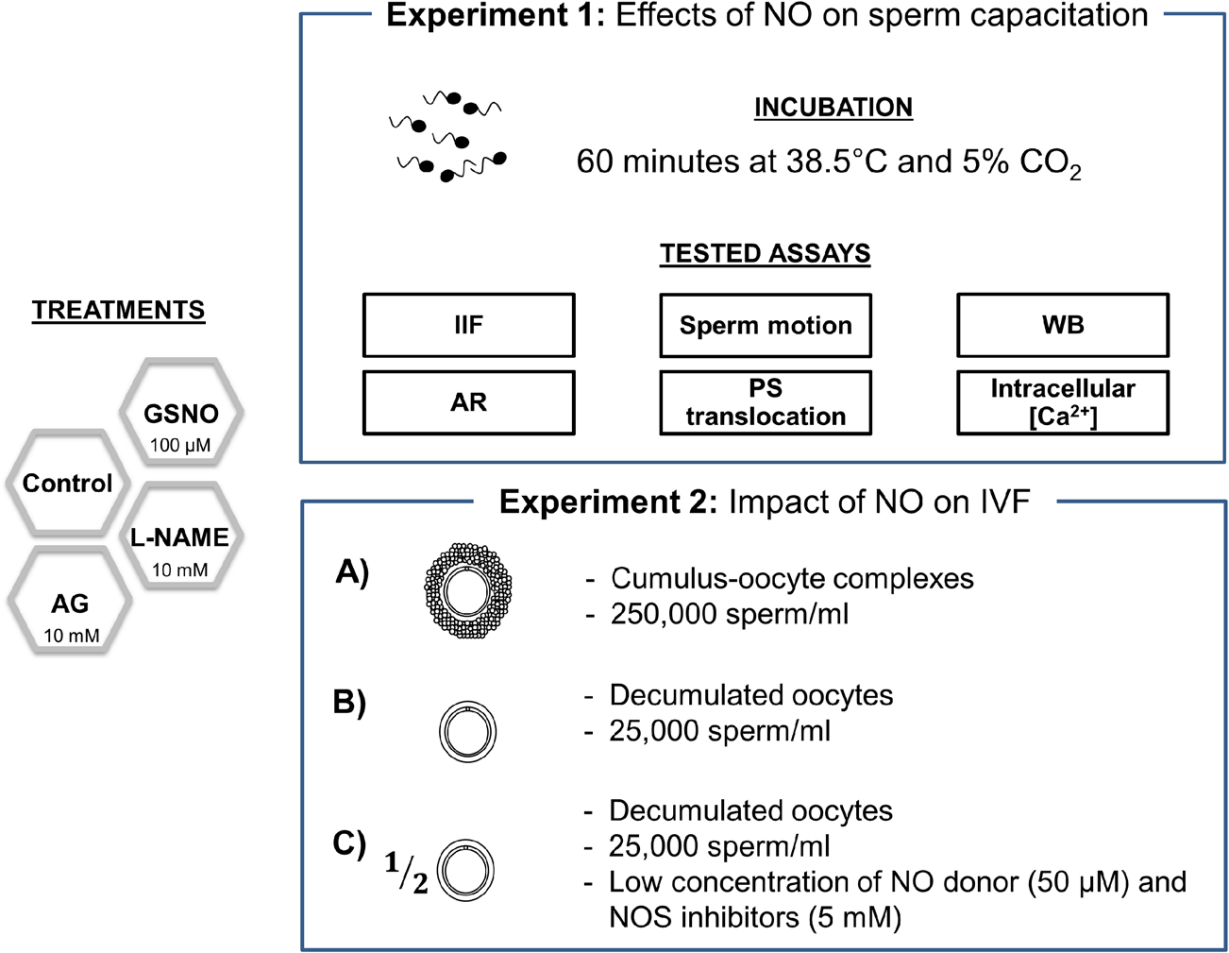
Analysis of the effects of a NO donor and two NOS inhibitors on sperm capacitation and in vitro fertilization. Experimental design. *Experiment 1*: Spermatozoa were incubated for 60 min in the presence or not of these supplements. After that, the following assays were used to evaluated sperm capacitation status: indirect immunofluorescence (IIF), motility assay, Western blotting (WB), acrosome reaction (AR), phosphatidylserine translocation (PS), and measurement of the intracellular calcium concentration; *Experiment 2*: The in vitro fertilization (IVF) was performed in the presence or not of the NO donor and NOS inhibitors, under three circumstances: (A) Intact cumulus-oocyte complexes and a sperm concentration of 250,000 spermatozoa/mL, (B) decumulated oocytes and a sperm concentration of 25,000 spermatozoa/mL, and (C) lower concentrations of the NO donor, NOS inhibitors, and spermatozoa. Neither the oocytes nor the spermatozoa were treated with the NO donor or NOS inhibitors before performing the IVF

To investigate how the NOS/NO system regulates sperm functionality (Fig. 1: experiment 1), sperm samples were incubated in TALP medium (capacitation medium) for 60 min at 38.5 °C and 5% CO_2_ with different treatments. Four experimental groups were established according to the treatment used: CONTROL: spermatozoa incubated in the absence of any treatment; GSNO: spermatozoa incubated in the presence of 100 μM S-nitrosoglutathione; L-NAME: spermatozoa incubated in the presence of 10 mM N^G^-nitro-L-arginine methyl ester hydrochloride; and AG: spermatozoa incubated in the presence of 10 mM aminoguanidine hemisulfate salt. These concentrations were chosen based on a literature review [3, 4, 25].

The experimental groups mentioned above were subjected to the following tests: indirect immunofluorescence (IIF) (to determine NOS localization and Tyr-P in situ), Western blotting (WB) (to evaluate the phosphorylation of PKA substrates and Tyr-P), acrosome reaction (AR) assay, PS translocation assay, and measurement of [Ca^2+^]_i_. However, to avoid sperm agglutination, which hinders cell detection by CASA systems, and since previous studies have reported 30 min sperm incubation under capacitation conditions were sufficient to observe changes in sperm motility parameters [26], this period of time was considered to be suitable to assess the effect of the NOS/NO system on sperm motion.

#### Experiment 2: impact of NO on IVF

To assess how the NOS/NO system modulates the IVF in porcine species, three experiments were performed (Fig. 1: experiment 2A, B, and C). All experiments were started using in vitro matured oocytes, and IVF was performed by adding to the medium the abovementioned NO donor and NOS inhibitors. As a control group, IVF was performed in the absence of any treatments. The spermatozoa employed during IVF were not previously treated with these supplements. The percentage of sperm penetration, the sperm number per oocyte, the number of sperm bound to the ZP, and the percentage of male pronucleus formation were determined in all experiments.

#### Experiment 2A: effects of NO on the interaction between spermatozoa and COCs

IVF was performed using COCs that were co-incubated with 2.5 × 10^5^ spermatozoa/mL. The GSNO was used at a concentration of 100 μM, whereas for the NOS inhibitors (L-NAME and AG) the concentration was 10 mM. This experiment was repeated five times, and a total of 549 oocytes were evaluated.

#### Experiment 2B: effects of NO on the interaction between spermatozoa and decumulated oocytes

Since the cumulus cells also produce NO [27], this second experiment was performed to investigate how the presence/absence of NO alters the interaction between sperm and decumulated oocytes. IVF was performed using the same concentrations of NO donor and NOS inhibitors as in experiment A. This experiment was repeated three times, and a total of 258 oocytes were evaluated.

#### Experiment 2C: effects of low NOS inhibitor concentration on the interaction between spermatozoa and decumulated oocytes

The latter assay was developed to evaluate whether there is a dose-dependent effect of the NO donor and NOS inhibitors. For this, IVF was performed using decumulated oocytes, 2.5 × 10^4^ spermatozoa/mL, and a lower concentration of NO donor and NOS inhibitors (50 μM GSNO and 5 mM for the inhibitors, respectively). This experiment was repeated three times, and a total of 351 oocytes were evaluated.

## Results

### Experiment 1: effects of NO on sperm capacitation

#### NOS localization

The three isoforms of NOS, neuronal (nNOS), endothelial (eNOS), and inducible NOS (iNOS), have been identified in different mammalian spermatozoa, including the boar [16]. However, to our knowledge no study has been performed to localize NOSs in porcine ejaculated spermatozoa. Therefore, we used IIF to identify the distribution of these enzymes.

**Fig. 2:**
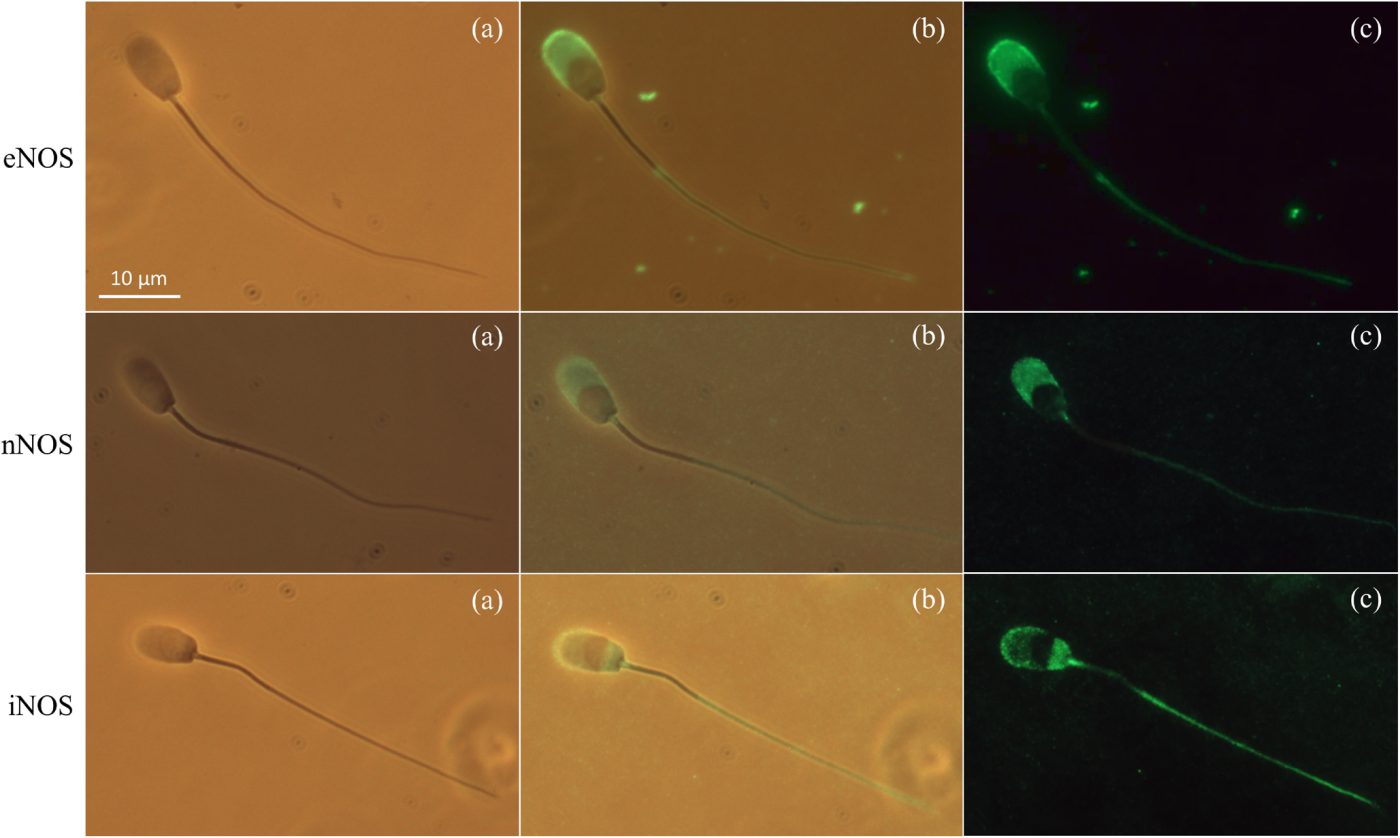
Localization of NOS isoforms by indirect immunofluorescence. Spermatozoa were fixed, permeabilized, and incubated with specific anti-eNOS, nNOS, and iNOS primary antibodies, together with a FITC-labeled secondary antibody and examined under an epifluorescence microscope at ×1000 magnification. Representative pictures are shown by phase-contrast microscopy (**a**), merging the phase-contrast image with the green fluorescence pattern (**b**) and for the immunofluorescent staining (**c**). The eNOS- and nNOS-associated fluorescence was identified in the sperm head region, with a lower staining in the principal and end pieces of the tail. The iNOS staining pattern was spread over the acrosomal, postacrosomal, and neck region but also in the principal and end pieces of the flagellum

The eNOS was identified in the acrosomal region, although a weak fluorescent signal was also registered in the principal and end piece of the flagellum (Fig. 2). Similarly, the nNOS-associated fluorescence was concentrated in the sperm head region, with a lower fluorescence in the principal and end piece of the flagellum (Fig. 2). Moreover, immunofluorescent iNOS staining was spread over the acrosomal, postacrosomal, and neck region but also in the principal and end pieces of the tail (Fig. 2).

#### Motility parameters

The role of NO in sperm motility is controversial, with studies suggesting both a beneficial [28, 29] or detrimental effect [30, 31].

When the CASA evaluation was performed in the present study, at 0 min incubation time (Table 1), none of the motility parameters showed statistical differences (*P* > 0.05). Later, at 30 min of incubation (Table 1), no differences were found for total motility, progressive motility, VCL, LIN, STR, WOB, ALH, or BCF. However, when the VSL was studied, CONTROL and GSNO groups (17.2 ± 1.3 and 17.9 ± 1.8, respectively) were found to be significantly different from AG (11.8 ± 0.8), but no differences were observed with L-NAME (16.4 ± 0.5). Continuing the sperm motion analysis, when we analyzed VAP at 30 min, both CONTROL and GSNO showed the highest values (26.6 ± 1.8 and 26.9 ± 2.6, respectively), which did not differ from the L-NAME group (25.5 ± 0.8) but were significantly different from AG (19.7 ± 0.9).

**Table 1:** Effects of NO on sperm motility parameters at 0 and 30 min of incubation

Incubation time	Treatment	Number	Total motility	Progressive motility	VCL	VSL	VAP	LIN	STR	WOB	ALH	BCF
0 min	CONTROL	6	86.8 ± 2.8	84.0 ± 3.2	95.1 ± 11.3	38.2 ± 4.9	59.7 ± 4.7	29.3 ± 9.3	39.2 ± 12.4	48.3 ± 15.1	3.1 ± 0.4	8.6 ± 0.3
	GSNO	6	83.6 ± 1.3	79.0 ± 2.2	90.4 ± 15.4	35.2 ± 3.8	54.6 ± 4.9	29.0 ± 9.2	39.2 ± 12.4	47.3 ± 14.8	3.1 ± 0.6	8.7 ± 0.3
	L-NAME	6	86.7 ± 2.8	82.4 ± 3.7	99.3 ± 17.5	36.6 ± 5.6	59.2 ± 6.1	26.7 ± 8.7	35.7 ± 11.6	49.0 ± 15.4	3.4 ± 0.7	8.2 ± 0.4
	AG	6	81.8 ± 3.2	77.4 ± 4.0	92.6 ± 13.1	41.0 ± 7.3	59.8 ± 7.0	29.9 ± 9.7	40.5 ± 13.1	47.5 ± 14.9	3.0 ± 0.5	8.8 ± 0.5
30 min	CONTROL	6	36.9 ± 5.6*	26.7 ± 4.9*	49.5 ± 7.0*	17.2 ± 1.3a*	26.6 ± 1.8a*	28.7 ± 9.0	41.5 ± 13.0	42.3 ± 13.3	2.0 ± 0.3*	5.5 ± 0.2*
	GSNO	6	41.0 ± 6.8*	29.9 ± 5.8*	50.9 ± 8.1*	17.9 ± 1.8a*	26.8 ± 2.6a*	29.0 ± 9.4	42.6 ± 13.4	41.9 ± 13.3	2.1 ± 0.3	5.7 ± 0.3*
	L-NAME	6	45.9 ± 7.7*	32.2 ± 6.8*	43.8 ± 4.2*	16.4 ± 0.5a,b*	25.5 ± 0.8a,b*	29.9 ± 9.4	42.1 ± 13.2	45.7 ± 14.3	1.8 ± 0.2*	6.0 ± 0.2*
	AG	6	30.6 ± 4.0*	21.3 ± 3.2*	43.6 ± 4.2*	11.8 ± 0.8b*	19.7 ± 0.9b*	24.1 ± 8.1	40.5 ± 12.9	36.5 ± 11.7	1.9 ± 0.2*	5.2 ± 0.3*

Total motility (%), progressive motility (%), VCL (curvilinear velocity, μm/s), VSL (straight-line velocity, μm/s), VAP (average path velocity, μm/s), LIN (linearity of the curvilinear trajectory, ratio of VSL/VCL, %), STR (straightness, ratio of VSL/VAP, %), WOB (wobble of the curvilinear trajectory, ratio of VAP/VCL, %), ALH (amplitude of lateral head displacement, μm), and BCF (beat cross-frequency, Hz). All data are expressed as the mean ± SEM. Different lowercase letters within the same column indicate statistical significance (*P* < 0.05)

*Number* number of replicates

*Statistical significance (*P* < 0.05) throughout the incubation time

When looking at the effect of incubation time on the CASA parameters, we observed that at 30 min of incubation, all treatments showed significant differences compared to their values at time zero for total motility, progressive motility, VCL, VSL, VAP, and BCF. The same difference was found among all treatments in ALH, except for GSNO. Finally, when we compared the values for LIN, STR, and WOB at *T* = 30 min, the different treatments did not statistically differ from their *T* = 0 min counterparts.

#### Protein kinase A substrates and tyrosine phosphorylation

The sperm capacitation process involves the early activation of protein kinases and the inactivation of protein phosphatases [32]. It has been reported that NO can modulate this process through the activation of the cAMP/PKA pathway [33] and it is directly involved in tyrosine phosphorylation by modulating both the cAMP/PKA and the extracellular signal-regulated kinase (ERK) pathways (reviewed by [34]).

**Fig. 3:**
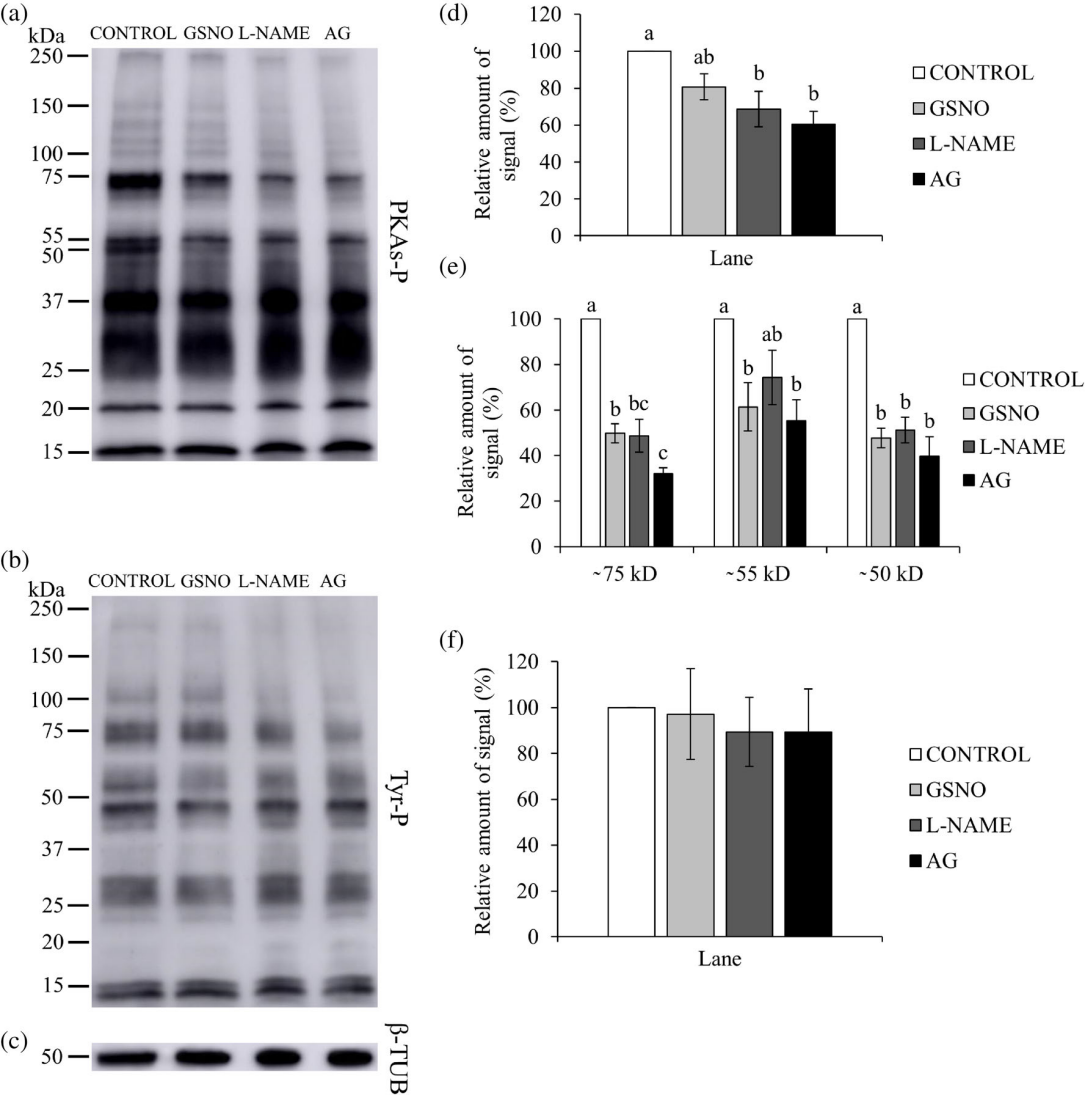
Effect of GSNO, L-NAME, and AG on PKA substrates (PKAs-P) and tyrosine phosphorylation (Tyr-P). Sperm were incubated for 60 min under capacitating conditions in the absence of any treatments (CONTROL) or in the presence of GSNO, a NO donor, and L-NAME and AG (both NOS inhibitors). (**a**, **b**) Sperm protein extracts were analyzed for phosphorylation by Western blotting using anti-PKAs-P or anti-Tyr-P as first antibodies, respectively. (**c**) β-Tubulin was used as a protein loading control. For signal quantification, each lane was normalized to its β-tubulin optical density value. (**d–f**) Relative amount of signal quantified in each membrane using ImageQuant TL v8.1 software for PKAs-P and Tyr-P, respectively. In the **d** and **f** bar charts, the lane axis represents the total amount of signal quantified in the four groups. Different letters (a, b, c) indicate statistically significant differences (*P* < 0.05) between groups

To determine the effects of the NO donor and NOS inhibitors on boar sperm capacitation, PKAs-P and Tyr-P were analyzed and quantified by WB (Fig. 3). Our results showed that the phosphorylation levels for PKAs-P were significantly lower when using the NOS inhibitors than in the CONTROL group (Fig. 3a, d), whereas the NO donor had no significant effect. Interestingly, the analysis of the relative optical density revealed the presence of three PKA substrate species of approximately 75, 55, and 50 kDa which seemed to possess a specific pattern of phosphorylation (Fig. 3a, e). In detail, the NO donor and NOS inhibitors lowered significantly the degree of phosphorylation in the ~ 75- and ~ 50-kDa species compared with their levels in the CONTROL (*P* < 0.05), but in the ~ 55-kDa species this effect was evident only when the capacitation took place in the presence of GSNO and AG (*P* < 0.05).

On the other hand, when considering the phosphorylation levels of tyrosine residues, no significant effects were observed in the presence of both the NO donor and NOS inhibitors (Fig. 3b, f).

#### Tyr-P detection by IIF

A crucial event involved in capacitation and the acquisition of fertilizing potential is protein Tyr-P [35]. Different sperm subpopulations were identified within a sample according to their degree of capacitation and hyperactivation (Table 2). No significant differences were found between groups with regard to the four Tyr-P patterns analyzed (*P* > 0.05).

**Table 2:** Effects of NO on the immunolocalization of protein Tyr-P

Treatment	Number	Pattern I (%)	Pattern II (%)	Pattern III (%)	Pattern IV (%)
CONTROL	8	10.8 ± 1.9	60.8 ± 9.2	28.5 ± 9.4	63.7 ± 9.7
GSNO	8	11.2 ± 2.3	53.2 ± 9.9	35.9 ± 9.1	64.3 ± 10.9
L-NAME	8	20.0 ± 6.6	46.4 ± 11.3	33.6 ± 11.3	63.0 ± 10.3
AG	8	10.9 ± 2.8	49.4 ± 11.3	39.8 ± 10.9	64.6 ± 8.2

Pattern I, low capacitation status (non-phosphorylated or head- and/or flagellum-phosphorylated spermatozoa); pattern II, medium capacitation status (equatorial segment or equatorial segment and flagellum-phosphorylated spermatozoa); pattern III, high capacitation status (equatorial segment and head- and/or flagellum-phosphorylated spermatozoa); pattern IV, flagellum phosphorylation independent of phosphorylation in other locations

*Number* number of replicates

#### AR assay

**Fig. 4:**
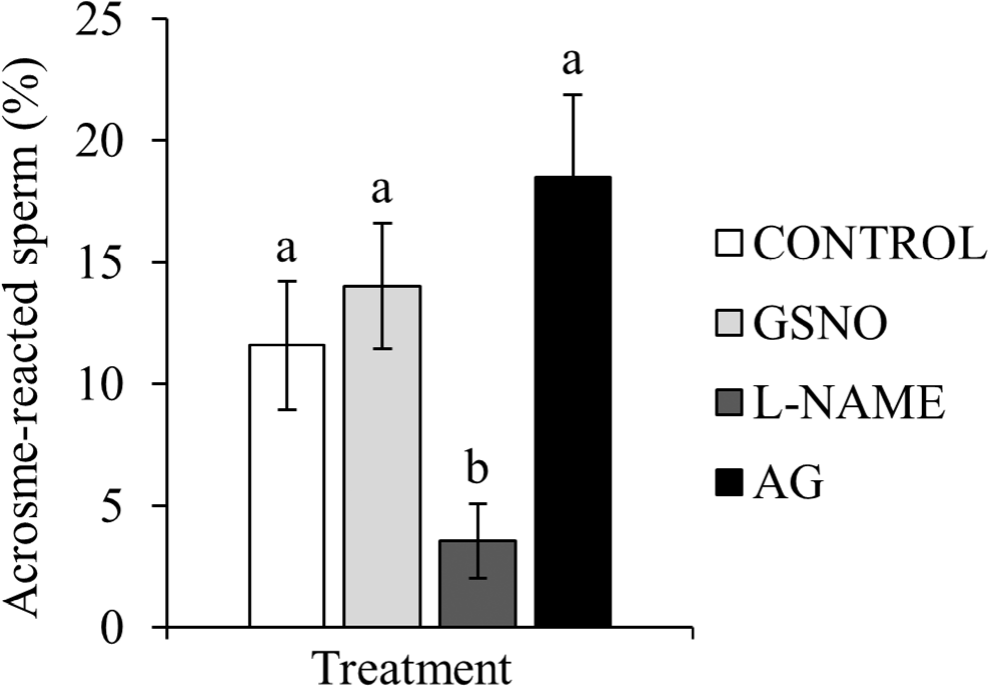
Effect of GSNO, L-NAME, and AG on the acrosome reaction. After being incubated in capacitating conditions for 60 min in the absence of any treatments (CONTROL) or in the presence of GSNO, a NO donor, and L-NAME and AG (both NOS inhibitors), the sperm were exposed to 3 ng/mL progesterone during another 30 min under the different experimental conditions. Next, the percentage of acrosome-reacted sperm was evaluated by PNA-FITC staining. Different letters (a, b) indicate statistically significant differences (*P* < 0.05) between groups

Progesterone is known to induce the acrosome reaction in capacitated sperm [36], so we determined how this process might be modulated by NO in boar spermatozoa. The results represented in Fig. 4 indicated that the GSNO and AG treatments did not influence the percentage of acrosome-reacted sperm when compared to the CONTROL. However, L-NAME reduced significantly this percentage (*P* < 0.05).

#### PS translocation

In boar spermatozoa, the capacitating agents had been shown to induce rapid changes in the membrane lipid architecture such as the external exposure of PS, which is also commonly recognized as a marker of apoptosis [37, 38]. As is considered that NO participates in both processes, we decided to investigate the involvement of NO in the PS translocation during sperm capacitation.

**Fig. 5:**
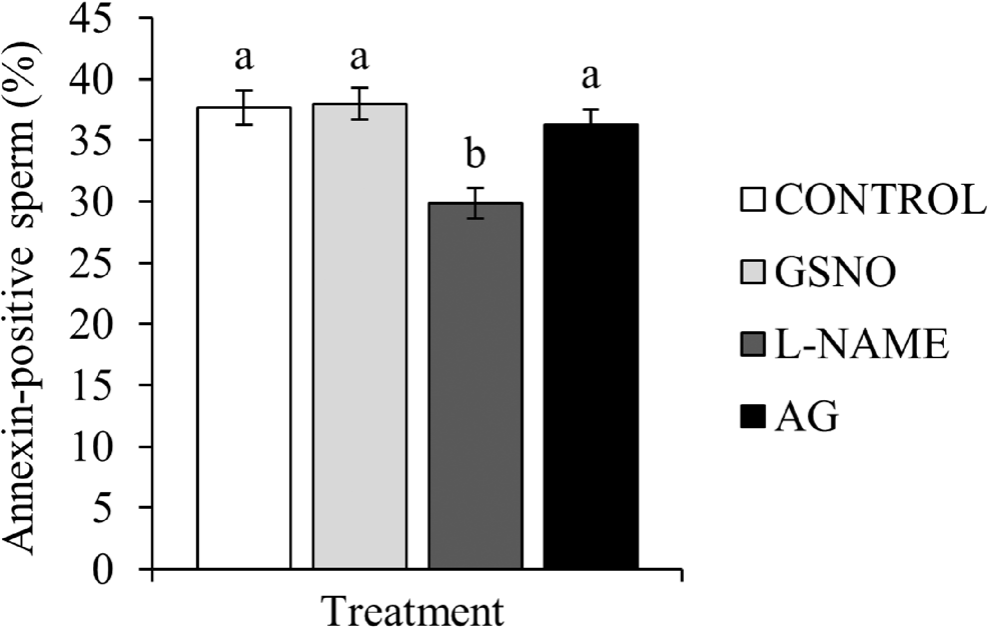
Effect of GSNO, L-NAME, and AG on PS translocation. Following incubation under capacitating conditions, the translocation of PS residues was analyzed with an Annexin V-Cy3™ Apoptosis Detection Kit. Different letters (a, b) indicate statistically significant differences (*P* < 0.05) between groups

The results (Fig. 5) showed that the NO donor had no significant effect on PS externalization. In fact, both the CONTROL and the GSNO groups reached similar levels of PS translocation (37.67% and 38%, respectively). On the other hand, when using the NOS inhibitors, a significant difference was observed only with L-NAME which had a lower PS level than both the GSNO and CONTROL groups (29.83%; *P* < 0.05). Sperm viability was higher than 50% in all the treatments (data not shown).

#### Determination of [Ca^2+^]_i_

**Fig. 6:**
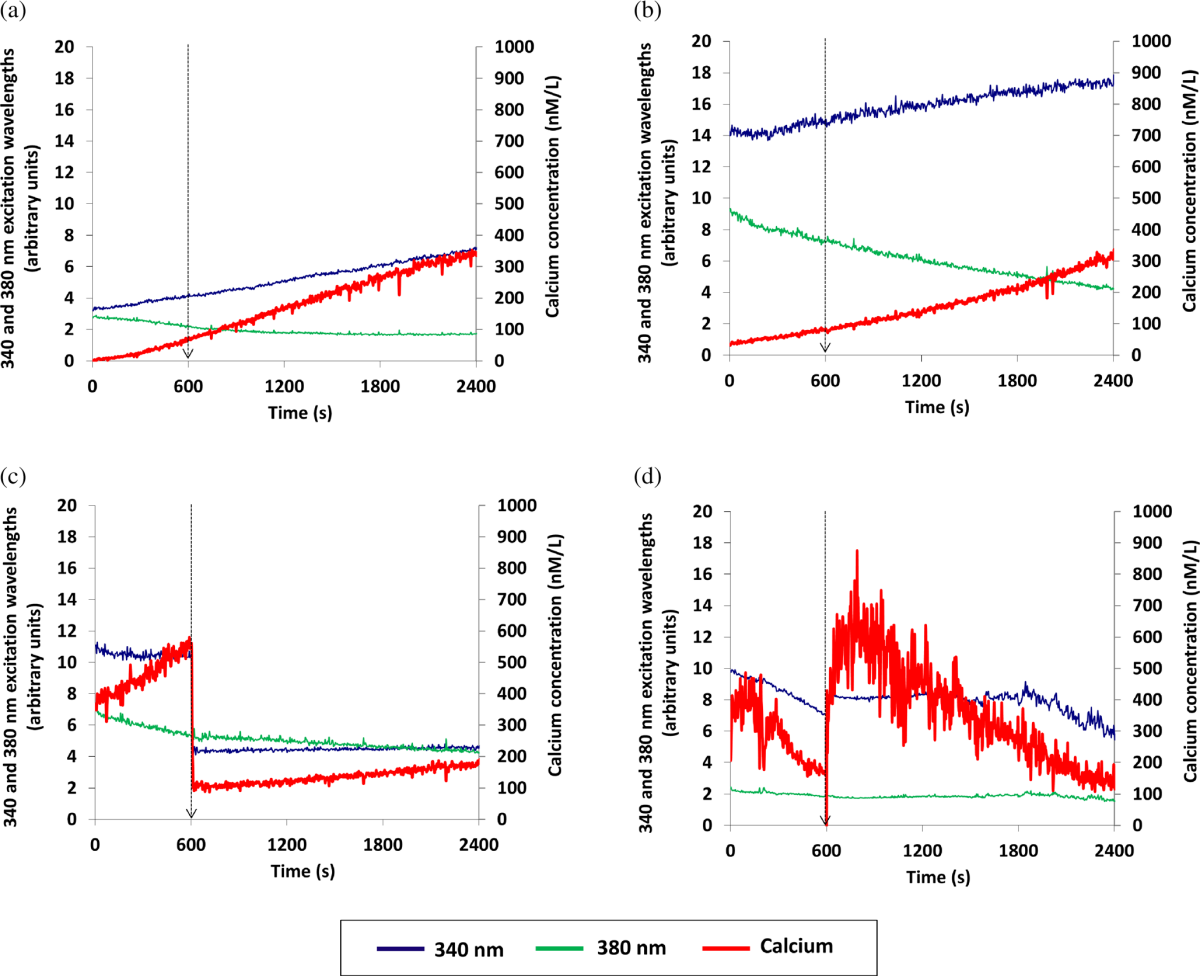
Intracellular calcium concentration. Graphs show the measurements collected from the different treatments: **a** control, **b** GSNO, **c** L-NAME, and **d** AG. The excitation wavelengths are shown with blue (340 nm) and green (380 nm) lines, while the intracellular calcium concentration is shown by the red line. Fluorescence was measured with the calcium indicator Fura-2/AM and monitored using a spectrofluorometer for 40 min. The system was stabilized for 10 min (dashed arrow) before adding or not the treatment

The regulation of Ca^2+^ is a fundamental step during the capacitation process [39]; therefore, we monitored its levels before and after our treatments (Fig. 6). During the period prior to the addition of treatments (600 s), Ca^2+^ intake increased throughout the incubation time. Treatment with GSNO did not affect [Ca^2+^]_i_ versus CONTROL. However, both inhibitors had an effect on the spermatozoa; in fact, results showed that L-NAME reduces abruptly the [Ca^2+^]_i_ at a basal level, while with AG the reduction is more gradual after its addition.

### Experiment 2: impact of NO on IVF

NO is one of the components of the environment where fertilization occurs and is generated by oviductal cells [40, 41], oocytes, and cumulus cells [9, 42] but also spermatozoa [15, 43, 44]. Besides, NO is necessary for sperm capacitation to occur [45]. However, it has been suggested that the sperm NO production is low and most likely these cells encounter sufficient NO levels to support capacitation inside the female genital tract [46]. For these reasons, we studied the effects of NO on the IVF parameters with and without cumulus cells.

#### Experiment 2A: effects of NO on the interaction between spermatozoa and COCs

The results (Table 3) showed that the inhibition of NO production affected all IVF parameters. The percentage of oocytes that had been fertilized in the presence of inhibitors decreased. The AG inhibitor reduced the IVF parameters, but these were higher than in the L-NAME group. As for the number of spermatozoa bound to the ZP and the mean number of spermatozoa per oocyte, we observed that they decreased both with the use of GSNO and with NOS inhibitors. In all the parameters analyzed, the NOS inhibitor with the greatest effect was L-NAME.

**Table 3:** Effects of NO during IVF with intact cumulus-oocyte complexes

Treatment	Number	Penetration (%)	Sperm/oocyte (*n*)	Sperm/ZP (*n*)	MPN formation (%)
CONTROL	139	100a	7.8 ± 0.3a	61.9 ± 3.5a	100
GSNO	128	93.0 ± 2.3a	6.5 ± 0.4a,b	40.8 ± 2.6b	100
L-NAME	136	1.5 ± 1c	1.5 ± 0.5c	13.8 ± 1.7c	100
AG	146	57.5 ± 4.1b	2.5 ± 0.2b,c	13.7 ± 1.3c	100

Lowercase letters in the same column denote significant differences (*P* < 0.05) between groups

*Number* number of evaluated oocytes per group, *MPN* male pronucleus formation

#### Experiment 2B: effects of NO on the interaction between spermatozoa and decumulated oocytes

To verify that the effect of the inhibitors was not influenced by the presence of cumulus cells, we decided to evaluate the IVF outcome with denuded oocytes. The obtained results are shown in Table 4, in which we can observe that there was no penetration when the IVF medium was supplemented with L-NAME and was very low when using AG. The addition of the NO donor to the fertilization medium had no significant effect on the percentage of penetration with respect to the CONTROL group. As for the spermatozoa adhered to the ZP, both the NO donor and the NOS inhibitors lowered this parameter when compared to the CONTROL.

**Table 4:** Effects of NO during IVF with denuded oocytes

Treatment	Number	Penetration (%)	Sperm/oocyte (*n*)	Sperm/ZP (%)	MPN formation (%)
CONTROL	61	98.4 ± 1.6a	8.3 ± 0.5a	41.2 ± 2.5a	98.3 ± 0.2a
GSNO	63	96.8 ± 2.2a	8.0 ± 0.5a	25.3 ± 1.3b	98.4 ± 0.2a
L-NAME	66	0	0	0.6 ± 0.1c	0
AG	68	17.6 ± 4.6c	1.8 ± 0.3b	5 ± 0.9c	100a

Lowercase letters in the same column denote significant differences (*P* < 0.05) between groups

*Number* number of evaluated oocytes per group, *MPN* male pronucleus formation

#### Experiment 2C: effects of low NOS inhibitors concentration on the interaction between spermatozoa and decumulated oocytes

Furthermore, we decided to analyze if IVF results would be modified by decreasing the inhibitor concentration, as well as that of spermatozoa (which synthesize NO). We observed (Table 5) that the inhibitors continued to have the same effect on all analyzed parameters. Also, with a lower sperm concentration (2.5 × 10^4^ spermatozoa/mL), the penetration percentage in the CONTROL and in the GSNO groups was lower than in our previous experiments, in which a 10-fold higher sperm concentration was used.

**Table 5:** Effects of NO during IVF with lower concentrations of sperm, NO donor, and NOS inhibitors

Treatment	Number	Penetration (%)	Sperm/oocyte (*n*)	Sperm/ZP (*n*)	MPN formation (%)
CONTROL	80	78.7 ± 4.6a	5.5 ± 0.9	42.8 ± 4.6a	96.8 ± 2.2
GSNO	76	77.6 ± 4.8a	7.2 ± 0.9	36.4 ± 3.9a	100
L-NAME	102	1.9 ± 1.4b	1.0 ± 0	5.0 ± 0.6b	100
AG	93	3.2 ± 1.8b	1.0 ± 0	4.9 ± 0.8b	100

Lowercase letters in the same column denote significant differences (*P* < 0.05) between groups

*Number* number of evaluated oocytes per group, *MPN* male pronucleus formation

## Discussion

The participation of the NOS/NO system in the reproductive function has been widely demonstrated [34]. NO has a dual role. Low amounts, generated under physiological conditions, seem to be beneficial for sperm functions [7, 28], but the excessive synthesis of NO, which takes place under in vitro fertilization conditions, could be detrimental for sperm function [47]. For that, the amount of NO in the fertilization media is variable and depends on the cumulus cells or sperm production, which could modify the capacitation process and the IVF outcome. The present study is, to the best of our knowledge, the first one to tackle both these aspects in porcine species, in the effort to obtain more insight on NO-mediated gamete interaction in vitro in this species.

NO synthesis takes place via L-arginine oxidation by three distinct NOS isoforms: neuronal (nNOS), endothelial (eNOS), also known as the constitutive isoforms, and the inducible NOS (iNOS) [48]. Numerous studies have been conducted to determine the presence and localization of these enzymes in sperm from several species [34] with slight differences between them [15, 44, 49]. However, the localization in boar sperm has not been described. We encountered a similar distribution between eNOS and nNOS, mostly in the sperm head region, whereas the immunofluorescent iNOS staining was spread on almost all sperm regions. This pattern could have a physiological significance, and it may suggest that the constitutive NOSs could be closely related to the activation of key pathways which leads to the capacitation [49], while the general distribution of the iNOS immunostaining might be connected to inflammatory processes in the male reproductive tract [50–52], rather than in the acquiring of the fertilization ability. We do not know if the NOS pattern exhibited by boar sperm changes during incubation in vitro, but this aspect should be addressed in future studies.

In the porcine species, research was focused mainly to address the involvement of NO in the promotion of capacitation [16, 53–55], lacking studies addressing the effect on sperm motility. In this sense, our results showed that despite that no differences were found at the beginning of the incubation, medium supplementation with AG, which selectively inhibits iNOS [56], significantly reduced VSL and VAP at 30 min of incubation. These results are completely opposite from the ones reported by Alizadeh et al. in varicocelized rats [57], where AG was shown to improve sperm motility and mitochondrial membrane potential. But both results are not comparable as their experimental design included an AG injection daily for 10 weeks, while we treated ejaculated sperm for 30 min. Nevertheless, it is worth noting that the reduction in these parameters has been linked to low breeding performance in porcine species [58]. In relation to the lack of a visible effect of the GSNO supplementation on sperm motility, this result agrees with Zini et al. [46] in human sperm, who demonstrated that a low concentration of a NO-releasing agent (0.1 mM), equal to the one we used, had no effect on the percentage of sperm motility or of hyperactivation. In regard to L-NAME, we did not find any significant difference in our experiment, while the addition of 10 mM L-NAME was reported to inhibit bull sperm progressive motility [25]. However, as some studies describe, this inhibitor is more likely to exert its effect on the inhibition of sperm capacitation rather than affecting sperm motility.

The phosphorylation levels of PKA substrates and tyrosine are known to be indicative of sperm capacitation status [26] and evidence confirms that NO regulates both serine/threonine [59] and tyrosine phosphorylation [60]. Our data suggest that the use of GSNO as a NO donor had no significant effect on the total level of phospho-PKA substrates (i.e., serine and threonine phosphorylation) and phosphorylation of tyrosine residues. On the contrary, Herrero and colleagues [60] have suggested that NO-releasing molecules might accelerate the capacitation process. In fact, when using sodium nitroprusside (SNP) during human sperm capacitation, an increase in tyrosine phosphorylation was observed. Similarly, Thundathil et al. [59] reported that the NO generated by spermine NONOate leads to an increase in the phosphorylation levels of the threonine-glutamine-tyrosine motif in two different human sperm proteins. However, we observed a specific phosphorylation pattern for three PKA substrate species, ~ 75, ~ 55, and ~ 50 kDa, which showed a lower degree of phosphorylation in the presence of GSNO. These data also seem to be in contrast with a previous work [61] and might be explained by the difference in the capacitation time (60 min in boar vs 240 min in human vs 90 min in mouse spermatozoa) and the species used, since the dynamics of serine/threonine phosphorylation are species-specific [62, 63]. On the other hand, our results showed that the inhibition of NO synthesis leads to a decrease in the levels of phospho-PKA substrates. This effect was more evident in the ~ 75- and ~ 50-kDa species. We speculate that these bands might contain proteins targeted for tyrosine phosphorylation, after they have been phosphorylated in serine/threonine by PKA in the presence of NO [64] to allow the correct development of the capacitation process.

NO is able to determine an increase in Tyr-P via the sGC-cGMP signaling pathway [34] at nanomolar levels [65] and the lack of NO due to NOS inhibition is correlated with lower levels of Tyr-P [60, 66]. However, according to our data, the NOS inhibitors had no effect on Tyr-P. It is possible that neither the NO donor nor the inhibitors used in our study were able to increase or lower Tyr-P because the low endogenous NO levels were enough to induce it [67]. This result is supported by our Tyr-P immunolocalization data, where no differences were observed between treatments.

At a molecular level, the AR shares a significant overlap with molecular events of capacitation [48] and both processes have been shown to be regulated by NO [46]. When incubating boar spermatozoa with exogenous NO, we did not observe any differences when compared to CONTROL. Other studies, however, report the NO donor’s ability to increase the percentage of acrosome-reacted sperm in boars [54] and different species (human [68], buffalo [69], and mouse [61]). This discrepancy might be explained by the different NO-releasing molecule used in these studies, which have different kinetics for NO generation [60]. Interestingly, when adding L-NAME to the incubation medium, the AR was significantly reduced in our study. This finding is consistent with previous studies in boar [16, 54], human [60], and hamster spermatozoa [70], which confirms that endogenous NO is necessary for spermatozoa to achieve their full fertilizing ability [60].

The translocation of PS is considered a physiological event during the capacitation process but also a sign of cellular damage [20, 38]. During sperm capacitation, the bicarbonate-stimulated protein phosphorylation pathway leads to the activation of phospholipid scramblase [37, 71] which results in the exposure of PS at the outer membrane surface [37]. Our results showed that the use of GSNO did not induce apoptotic-like changes in sperm when compared to CONTROL. This contrasts the findings of Moran et al. [72], and the reason might be the different methodological approach, namely, the use of a different NO-releasing compound and its concentration (100 μM GSNO vs 400 μM SNP). It has been reported that an increase in Annexin-positive spermatozoa is related to capacitation in boar semen [72] and that NOS inhibitors prevent capacitation [70]. This is in accordance with our observations regarding the NOS inhibitor L-NAME, which lowered significantly the percentage of Annexin-positive sperm.

In sperm, [Ca^2+^]_i_ changes through two routes, either Ca^2+^ ions are released from internal stores or transported into the cell by sperm-specific membrane channels [62, 73]. Previous studies have shown that NO can interact with different Ca^2+^ routes [74–76] also in spermatozoa [27]. In this sense, we have investigated how the NOS/NO system regulates [Ca^2+^]_i_ in porcine sperm. The results showed changes only when NOS inhibitors were used, L-NAME having the most potent effect. We hypothesized that in its presence, Ca^2+^ ions get expelled quickly from spermatozoa mainly through the Ca^2+^ efflux pump, plasma membrane calcium ATPase 4 (PMCA4) [77, 78], which is known to regulate NO signaling by downregulating the NOSs in murine sperm [77]. When using L-NAME, the PMCA4-NOS interaction might not have taken place, which in turn might have led PMCA4 to extrude the cytosolic Ca^2+^. Further experiments are needed to test this hypothesis.

Although it also affects the rest of the NOS isoforms, AG preferentially inhibits the iNOS isoform [79], which could explain why the reduction of [Ca^2+^]_i_ when adding this inhibitor is not as pronounced as it is with L-NAME. Our results suggest that in the beginning the Ca^2+^ output is compensated by the Ca^2+^ which comes from the internal stores causing the increase in [Ca^2+^]_i_. Once the internal stores are empty, [Ca^2+^]_i_ begins to decrease until reaching levels similar to those obtained with L-NAME. No significant differences were observed in relation to the GSNO supplementation, suggesting that NO contributes to the gradual increase in [Ca^2+^]_i_. This effect may be observed as a consequence of NO-mediated S-nitrosylation on sperm Ca^2+^ stores such as the ryanodine receptors [27, 80, 81]. Clearly, more experiments will be needed to confirm these data.

We have shown that the inhibition of NO synthesis, mainly by L-NAME, affects protein phosphorylation, acrosome reaction, and Ca^2+^ fluxes. However, the best test that indirectly evaluates sperm capacitation is the IVF [82], because only fully capacitated sperm can bind to the ZP, undergo acrosome reaction, and penetrate the oocyte’s plasma membrane. Consequently, we studied the modulation of sperm capacitation by NO in an IVF system under three circumstances: (i) IVF with cumulus-oocyte complexes, (ii) IVF with denuded oocytes, and (iii) denuded oocytes with reduced concentrations of NO donor and inhibitors. The results showed that under these three circumstances the tendency was the same; that is, in the presence of NOS inhibitors, the number of spermatozoa adhered to the ZP and the percentage of penetrated oocytes, and the mean number of spermatozoa per penetrated oocyte decreased. In addition, this effect was more pronounced when the L-NAME inhibitor was used.

Although it was proved that spermatozoa can synthesize NO, the evidence that its synthesis is sufficient to be physiologically important is not very clear [83]. For this reason, the first part of our IVF experiments was done with oocytes together with cumulus cells which generate significant amounts of NO and, therefore, participate in the processes of capacitation and fertilization [27]. Under these circumstances, we observed that both NOS inhibitors (L-NAME and AG) decreased the penetration rate but in a different way: AG reduced this parameter to half versus CONTROL, while L-NAME reduced it almost to zero. This may lead us to believe that the inhibitory effect of AG on NO production from cumulus cells or spermatozoa is not total since AG is a less potent inhibitor of the constitutive isoforms [79]. For this reason, enough NO could still be produced by the constitutive isoforms thus allowing the capacitation in some spermatozoa.

In 2008, Hou et al. [54] observed that the addition of L-NAME inhibited NO production by 30–40%, impairing the ability of spermatozoa to undergo the acrosome reaction. However, in our experiment, the addition of L-NAME decreased the penetration rate almost to zero, the AR levels being also significantly reduced. Therefore, in these conditions we could assume that NO synthesis was almost completely abolished, which might be explained by the fact that the inhibitor concentration used in our study was higher than the one used by Hou and colleagues.

Since cumulus cells could be differently sensible to NOS inhibitors, we considered performing IVF using denuded oocytes. The results showed a big decrease in the penetration rate with the AG inhibitor and zero penetration with L-NAME, so in the first experiment, the cumulus cells even in the presence of inhibitors were able to generate NO to allow sperm capacitation and fertilization. Finally, with the purpose of checking if the results previously obtained were due to the high concentration of NO donor and NOS inhibitors or a high number of sperm in the medium, we decided to reduce these parameters. We observed that the penetration in the CONTROL and GSNO groups decreased, but it did not increase in the inhibitor groups. We can assume that NO sperm production continued being abolished. On the other hand, Leal et al. [25] obtained a penetration rate of 70% in bovine with the same L-NAME concentration. Perhaps in this species, the constitutive NOSs in sperm are less sensible to this inhibitor. In other species, such as human [10] or mouse [8], it has been shown that the inhibitor effects of L-NAME were dose-dependent and the oocyte penetration could be affected even without modifications in the sperm capacitation parameters [10]. In contrast to Francavilla et al. [10], who observed that constitutive NOS play a role in the human sperm’s capacity to fuse with oocyte but not in the ZP binding, our results showed that even though the binding was not completely abolished, it decreased, so we can assume that the primary binding is less affected by NO absence. Interestingly, the NO donor GSNO lowered significantly the number of sperm bound to the ZP when compared with the CONTROL in either presence or absence of cumulus cells. A similar finding was reported by Wu et al. [84], suggesting that physiologic levels of NO are required for the binding process.

## Conclusions

During the past years, many studies focused on the role of NO in the physiology of reproduction. However, a clear investigation addressing the ability of the NOS/NO duo to modulate/affect in vitro both sperm capacitation and their interaction with the oocyte has been lacking in boars. The present work strongly suggests the importance of a delicate regulation of NOS enzymes during capacitation and IVF. NOS distribution, evidenced here for the first time in porcine spermatozoa, might be linked to key factors in the acquisition of a full fertilizing ability, such as protein phosphorylation, acrosome reaction, and intracellular Ca^2+^ fluxes. Our data shows how both sperm and cumulus-oocyte derived NO is required for successful IVF. Nevertheless, further studies should provide more information on these mechanisms in the attempt to solve IVF issues in porcine species, such as polyspermy.

## Electronic supplementary material

ESM 1 (DOCX 677 kb)
