# Supplementary material for: Regulation of boar sperm functionality by the nitric oxide synthase/nitric oxide system
Source: J Assist Reprod Genet. 2019 Jul 19;36(8):1721–36. doi: 10.1007/s10815-019-01526-6 (PMC6707978; doi:10.1007/s10815-019-01526-6)
Supplement: Supplementary file 1 — (DOCX 677 kb) [file 10815_2019_1526_MOESM1_ESM.docx]

**Regulation of boar sperm functionality by the nitric oxide synthase/nitric oxide system**

**Short running title:** Nitric Oxide Synthases - function in boar sperm.

**Authors and affiliations:** Florentin-Daniel Staicu ^a,b^, Rebeca Lopez-Úbeda ^b,c^, Jon Romero-Aguirregomezcorta ^a,b,d^, Juan Carlos Martínez-Soto ^b,e^ and Carmen Matás Parra ^a,b,^[[1]](#footnote-1)^*^

^a^ Department of Physiology, Veterinary Faculty, University of Murcia, International Excellence Campus for Higher Education and Research (Campus Mare Nostrum), Murcia, Spain.

^b^ Institute for Biomedical Research of Murcia (IMIB), Murcia, Spain.

^c^ Department of Cell Biology and Histology, Faculty of Medicine, University of Murcia, International Excellence Campus for Higher Education and Research (Campus Mare Nostrum), Murcia, Spain.

^d^ Department of Physiology, Faculty of Medicine and Nursing, University of the Basque Country (UPV/EHU), Bizkaia, Spain.

^e^ IVI-RMA Global, Murcia, Spain.


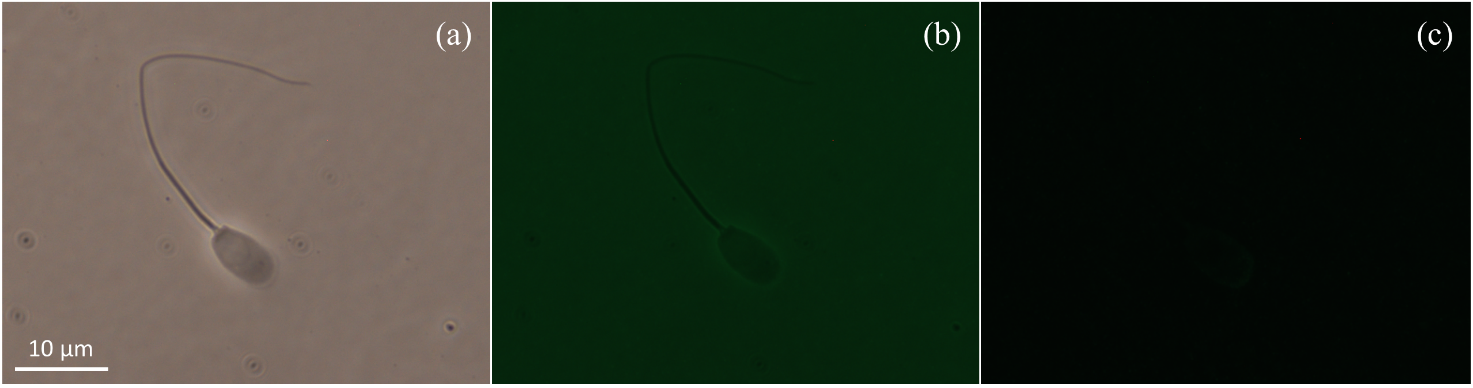


**Fig. 7 Negative control of the indirect immunofluorescence technique applied for NOSs localization.** Spermatozoa were fixed, permeabilized, incubated with a FITC-labeled secondary antibody and examined under an epifluorescence microscope at ×1000 magnification. Representative pictures are shown by phase-contrast microscopy (**a**), merging the phase-contrast image with the green fluorescence pattern (**b**) and for the immunofluorescent staining (**c**). No non-specific staining was observed


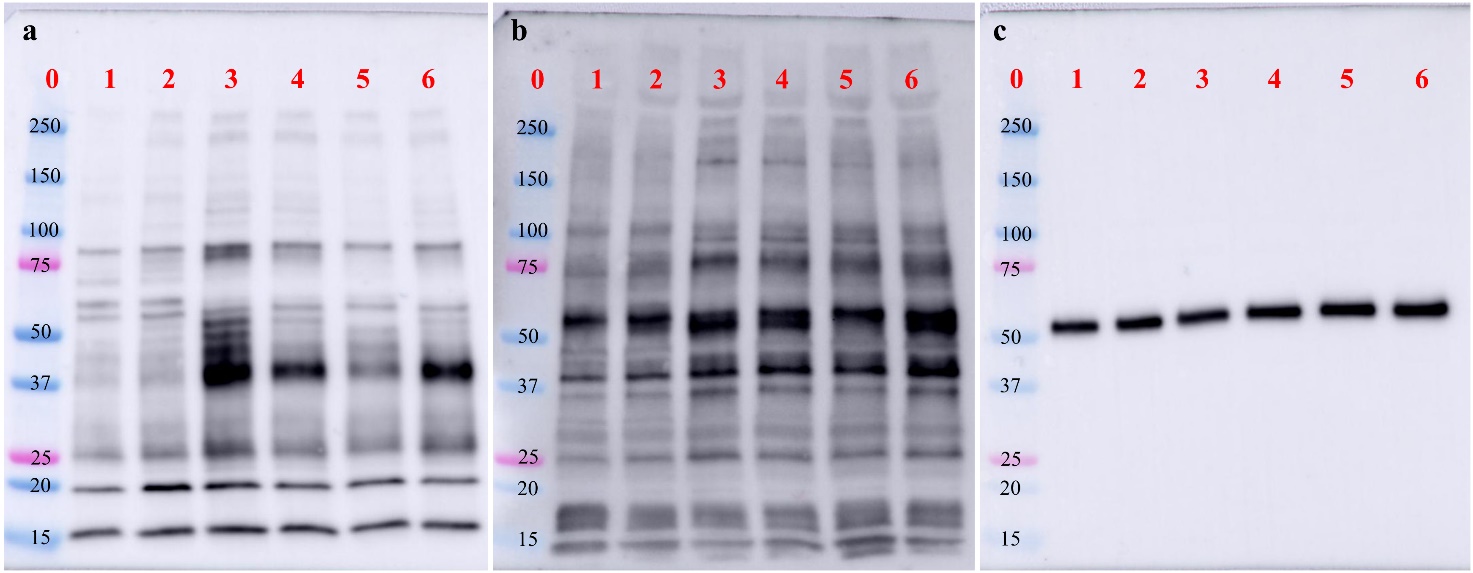


**Fig. 8 Representative membranes for phospho-PKA substrates (a), tyrosine phosphorylation (b) and β-tubulin (c).** Red numbers indicate the content of each lane. (**0**) Precision Plus Protein^TM^ Dual Color Standards (Bio-Rad, Madrid, Spain, #1610374). Molecular weights are expressed in kilodaltons. (1–6) Immunoblotted protein extracts from spermatozoa incubated under different experimental conditions, as follows: (1) Dulbecco’s phosphate-buffered saline without calcium chloride and magnesium chloride, time 0 h. (2) TALP medium, time 0 h. (3) CONTROL: TALP medium, time 1 h. (4) GSNO: TALP medium supplemented with 100 µM S-nitrosoglutathione, time 1 h. (5) L-NAME: TALP medium supplemented with 10 mM N^G^-nitro-L-arginine methyl ester hydrochloride, time 1 h. (6) AG: TALP medium supplemented with 10 mM aminoguanidine hemisulfate salt, time 1 h

1. * Corresponding author: Carmen Matás Parra, E-mail: cmatas@um.es; Tel.: +34-868-88-7256. [↑](#footnote-ref-1)
